# The Spectrum of Solitary Benign Splenic Lesions—Imaging Clues for a Noninvasive Diagnosis

**DOI:** 10.3390/diagnostics13122120

**Published:** 2023-06-20

**Authors:** Sofia Gourtsoyianni, Michael Laniado, Luis Ros-Mendoza, Giancarlo Mansueto, Giulia A. Zamboni

**Affiliations:** 1 1st Department of Radiology, School of Medicine, National and Kapodistrian University of Athens, Areteion Hospital, 76, Vas. Sophias Ave., 11528 Athens, Greece; sgty76@gmail.com; 2 Institute and Policlinic for Diagnostic and Interventional Radiology, University Hospital Carl Gustav Carus, TU Dresden, Fetscherstraße 74, 01307 Dresden, Germany; michael@laniado.de; 3 Department of Radiology, Miguel Servet University Hospital, Paseo Isabel la Católica 1-3, 50009 Zaragoza, Spain; lhrosmendoza@gmail.com; 4 Istituto di Radiologia, DAI Patologia e Diagnostica, Policlinico GB Rossi, AOUI Verona, 37134 Verona, Italy; giancarlo.mansueto@univr.it

**Keywords:** spleen, benign, solitary, MRI, CT

## Abstract

Cross-sectional imaging of the upper abdomen, especially if intravenous contrast has been administered, will most likely reveal any acute or chronic disease harbored in the spleen. Unless imaging is performed with the specific purpose of evaluating the spleen or characterizing a known splenic lesion, incidentally discovered splenic lesions pose a small challenge. Solitary benign splenic lesions include cysts, hemangiomas, sclerosing angiomatous nodular transformation (SANT), hamartomas, and abscesses, among others. Sarcoidosis and tuberculosis, although predominantly diffuse micronodular disease processes, may also present as a solitary splenic mass lesion. In addition, infarction and rupture, both traumatic and spontaneous, may take place in the spleen. This review aims to describe the imaging features of the most common benign focal splenic lesions, with emphasis on the imaging findings as these are encountered on routine cross-sectional imaging from a multicenter pool of cases that, coupled with clinical information, can allow a definite diagnosis.

## 1. Introduction

The spleen, often referred to as the forgotten organ, serves in adulthood as a filter for blood cells with additional important immune functions. As it is not essential for the preservation of vital life functions, it often receives limited attention from clinicians. Nevertheless, a wide range of diseases can affect the spleen, which might present as focal lesions on routine cross-sectional imaging, classified into six categories (Table 1).

Incidental splenic lesions, defined as lesions detected by imaging performed for a reason unrelated to the spleen, have not been evaluated in great detail and may equally excite and puzzle the reporting Radiologist. In a population-based whole-body magnetic resonance imaging (MRI) study of 2500 healthy individuals, 31.5% had incidental findings of potential clinical relevance, but only 12 involved the spleen with a reported likelihood of malignancy of only 1% [1]. Another study performed on trauma victims reported an incidence of less than 2% for splenic granulomas, hemangiomas, cysts, and abscesses, proving that most incidentally detected isolated splenic lesions are benign [2].

Unfortunately, due to overlapping imaging features, differentiation of benign from malignant splenic lesions may be challenging. Clinical history plays a pivotal role in incidental splenic lesion characterization, including pain related to the spleen, body temperature, immune status, and history of recent trauma. Significant predictors of malignant splenic lesions are the solid nature of the mass, lymph node enlargement, and/or the presence of underlying malignancy [3].

In this paper, cross-sectional imaging findings of the most common benign focal splenic lesions are reviewed, highlighting their key differences, as all typically present as hypodense on CT, with low signal intensity (SI) on T1 weighted images (WI) and most of the time hyperintense on T2-WI, exceptions listed in Table 2.

## 2. Normal Splenic Appearances with Different Imaging Modalities

### 2.1. US

Ultrasonography (US) is frequently the first imaging modality to assess the spleen because of its high diffusion and lack of ionizing radiation. In the hands of an experienced examiner, it has high reliability. On US, the splenic parenchyma is typically homogeneous, with finely textured internal echoes, and is slightly more echoic than the normal renal cortex, isoechoic to slightly hyperechoic compared to the liver parenchyma and hypoechoic compared to the pancreatic parenchyma. Color Doppler can be useful in the evaluation of vascular pathology in the splenic hilum. US can easily detect focal splenic lesions, but in most cases, these have a nonspecific appearance, and the diagnostic accuracy in the characterization is limited (30–75%). However, this has been reported to improve with the use of contrast-enhanced US [4]. The contrast medium currently available in Europe is Sonovue^®^ (Bracco, Milan, Italy), an intravascular agent consisting of microbubbles (1–7 μm) containing Sulfur hexafluoride encapsulated by a phospholipid shell. The microbubbles remain inside the vessels for up to 7 min, after which they dissolve, with the gas exhaled through the lungs, while the shell gets metabolized, primarily by the liver. In order to avoid microbubble rupture under injection pressure, a needle with a diameter of 23 Gauge or larger must be used in adults to administer a bolus of 2 mL of ultrasonographic contrast medium, followed by approximately 10 mL of saline solution through an antecubital vein. A nonenhanced US examination is performed to identify the best scan view followed by arterial phase imaging at 12–20 s post-injection, during which the normal spleen demonstrates a zebra pattern, such as the one in CECT. During this phase, arterial vascular injuries may be detected. The venous phase starts after 40–60 s post-injection, being the optimal phase for organ injury detection. The healthy splenic parenchyma demonstrates a homogeneous enhancement for an extended period, approximately 5–7 min [5].

### 2.2. CT

On unenhanced CT, the spleen is homogeneous, has attenuation values ranging between 40 HU and 60 HU, and is hypodense or isodense to the liver parenchyma. On unenhanced images, calcifications can be easily detected. After intravenous contrast administration, the normal splenic parenchyma has a variable pattern of enhancement in the early and late arterial phases. The enhancement patterns include serpentine or mottled appearance and focal and diffuse heterogeneity because of variable flow rates through circulation in the white and red pulp, giving the typical “zebra-like” enhancement. In the portal-venous phase, the enhancement of the normal parenchyma becomes homogeneous.

### 2.3. MRI

Magnetic Resonance (MR), using a combination of T2-weighted, gradient-echo, and multiphasic contrast-enhanced imaging, provides superior lesion characterization compared with CT or US. On T1-weighted images (WI), the MR signal intensity of the splenic tissue is homogeneously low and equal to slightly less than that of the liver and muscle. On T2-WI, signal intensity is usually higher than that of the liver parenchyma. The spleen remains the brightest abdominal organ in T2-weighted images, even though signal intensity varies with patient age. Because the white pulp is not yet matured in the newborn, signal intensity is usually more hypointense than the normal liver parenchyma on T2-WI and more isointense on T1-WI. Splenic imaging characteristics evolve to the normal adult pattern within the first months of life. Νormal splenic parenchyma tends to demonstrate a homogenously restricted diffusion on Diffusion-weighted imaging (DWI), i.e., high signal intensity on the highest b value obtained and low signal intensity on the corresponding ADC map [6]. Splenic lesions with a hyper- or isointense signal on high b-value DWI images (e.g., b 800) and iso- or hypointense signal on the corresponding ADC map compared to the normal splenic parenchyma are more likely malignant. Therefore, the addition of DWI to conventional MRI improves the prediction of malignant splenic lesions [7]. After dynamic administration of gadolinium contrast agents, the enhancement pattern of the spleen is similar to the contrast enhancement pattern on CT, appearing inhomogeneous in the arterial phase and homogeneous in the portal venous and equilibrium phases. A splenic lesion with low signal intensity on 3-min delayed images combined with arterial hypervascularity on dynamic MRI may predict the malignant nature of the splenic lesion, while the 3-min delayed phase low signal intensity and the presence of restricted diffusion raise the diagnostic performance for the discrimination of focal splenic lesions [8].

### 2.4. Nuclear Medicine

Nuclear medicine offers different radiopharmaceutical tracers to be used with conventional scintigraphy or Positron Emission Tomography (PET). Since Technetium Tc99m sulfur colloid accumulates in the liver and spleen through the uptake by the reticuloendothelial system, scintigraphy can be used to localize ectopic splenic tissue in cases of intrapancreatic accessory spleen, splenosis, or wandering spleen. PET with [18F]-Fluoro Deoxy Glucose (18F-FDG-PET) shows lower metabolism and tracer uptake of the normal spleen compared to the liver [9]. Diffusely increased splenic uptake is observed after administration of granulocyte colony-stimulating factor [10], but also in some cases of neoplastic (e.g., lymphomatous involvement) and inflammatory diseases (e.g., HIV, sarcoidosis) [11]. Focal tracer accumulation in the spleen occurs secondary to the presence of neoplastic or inflammatory lesions [12].

## 3. Lesion-Specific Imaging Characteristics

**Figure 1:**
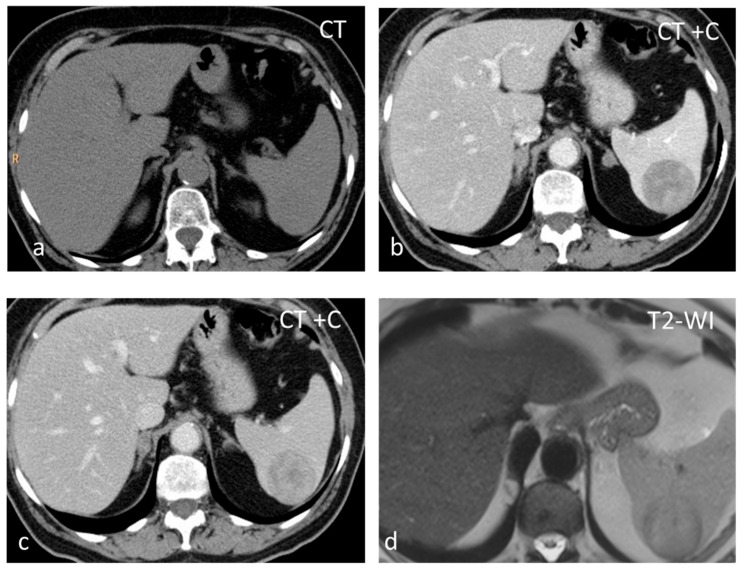
Large solid lesion, in keeping with an inflammatory myofibroblastic tumor, slightly hypodense in the non-contrast CT scan (**a**), depicting slightly heterogenous/patchy gradual enhancement pattern post-contrast enhancement (**b**,**c**). The lesion appears iso– to hypointense on T2-weighted MRI, more conspicuous than in the non-contrast CT scan (**d**).

***Inflammatory myofibroblastic tumor (IMT),*** formerly known as inflammatory pseudotumor, is an uncommon benign splenic lesion. It has been described in virtually all major organs with a few exceptions. In the liver and spleen, inflammatory pseudotumor is possibly linked to Epstein–Barr infection [13]. The prevalence is similar in both sexes, with a peak incidence in middle age. The lesions reported are usually large, measuring > 10 cm. It is composed of a combination of inflammatory and myofibroblastic spindle cells. Usually an incidental finding, it is included in the differential diagnosis of malignant splenic lesions, although currently, the World Health Organization classification of soft tissue tumors places IMTs in an intermediate category between benign and malignant, with metastases in less than 5% of extrasplenic cases [14,15]. In the US, inflammatory myofibroblastic tumors appear as solid hypoechoic masses. CT shows hypoattenuating hypoenhancing lesions, and stellate central calcifications seen on CT scans make the diagnosis very likely [16]. They present as hypointense masses both on T1-WI and T2-WI, with slow delayed enhancement (Figure 1) [17]. The diagnosis can be confirmed reliably only by histopathological and immunohistochemical evaluations. Although recurrence and metastatization have not been described for splenic inflammatory myofibroblastic tumors, patients must be followed up as these are considered tumors with intermediate malignant potential [14,17].

**Figure 2:**
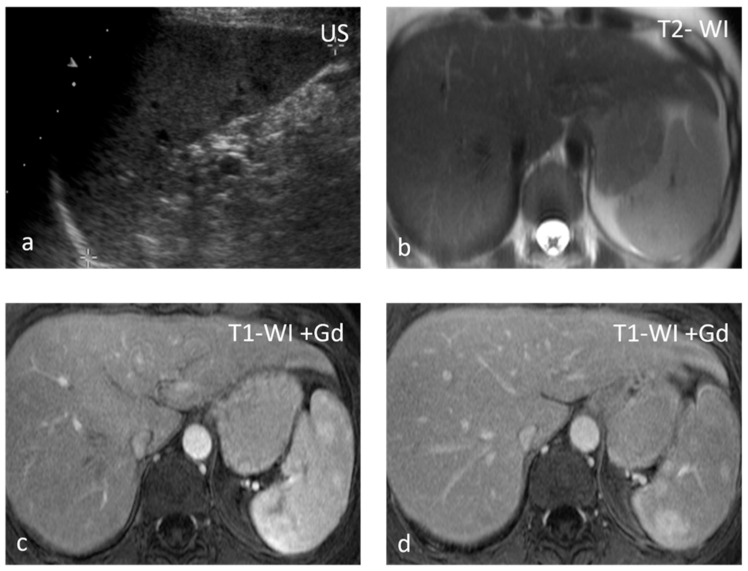
Sarcoidosis. US shows multiple hypoechoic nodules (**a**). The nodules appear slightly hypointense on T2 WI (**b**) and show mild delayed enhancement (**c**,**d**).

***Sarcoidosis*** is a multisystem disease characterized by the presence of non-caseating granulomas. One-third of patients presenting with splenic sarcoidosis findings have normal chest radiography [18]. Splenic involvement has been reported in about 40% of cases of multisystem sarcoidosis, but isolated sarcoidosis of the spleen is extremely rare [19]. It may present either as splenomegaly or with multiple nodules, whereas a solitary splenic lesion is very rare. Patients with diffuse splenic granulomas have a worse prognosis in terms of persistent chronic sarcoidosis than patients without splenic involvement or patients with limited splenic disease [20]. Nodules appear hypodense on CT and have low SI both on T1-WI and T2-WI, with minimal delayed enhancement [21] (Figure 2). Nodules are best seen on T2-WI with fat saturation and on early gadolinium-enhanced T1–WI. MRI is said to be able to monitor disease activity, as during active inflammation, nodules demonstrate T2-WI hyperintensity due to edema and high vascular permeability, as well as restricted diffusion [22]. The main differential diagnosis includes infections, especially tuberculosis, and malignancies, especially lymphoma. The final diagnosis is based on three main criteria: a compatible presentation, the evidence of non-caseating granulomas on histological examination, and the exclusion of any alternative diagnosis [23].

**Figure 3:**
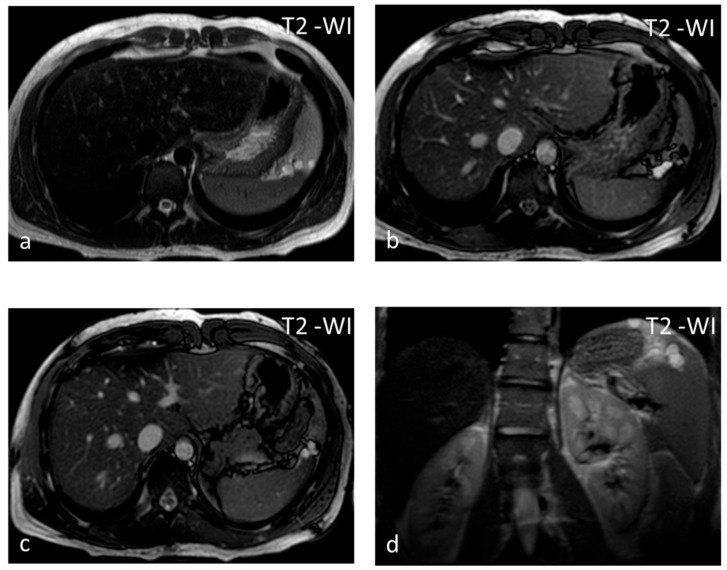
Lymphangiomas. Multiple, subcapsular, hyperintense lesions, one of which appears multiloculated with thin septa, are seen on axial T2-WI images (**a**–**c**) and coronal plane T2-WI image (**d**).

***Lymphangioma***, a vascular lesion like hemangioma, is a rare benign lesion that is commonly seen in children and exceptional in adults. In 60% of cases, the diagnosis is made before the age of 1 year. Lymphangioma is commonly subcapsular and may have satellite lesions [24]. Similarly to hemangiomas, lymphangiomas can involve the spleen exclusively, or they may be part of generalized angiomatosis, with lymphangiomas or hemangiomas involving several organs in the body [25]. US shows a rounded, well-defined hypoechoic lesion, possibly with internal septations and intralocular echogenic debris [26]. On CT, lymphangiomas appear as single or multiple thin-walled hypodense lesions with no enhancement in a typical subcapsular location [26]. Peripheral wall calcifications can be present. The lesion has typical benign cystic MRI features and may be multiloculated with hypointense thin septa (Figure 3), which may show enhancement. The management of choice for symptomatic, i.e., presenting with left upper quadrant pain and/or splenomegaly, lymphangiomas is splenectomy, as delay in therapeutic intervention can lead to life-threatening complications [24].

**Figure 4:**
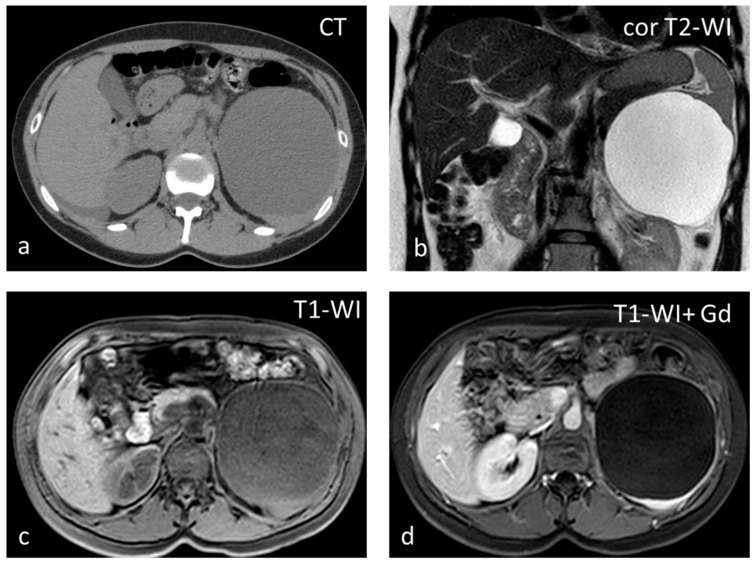
Large splenic cyst appearing homogeneously hypodense on non-contrast CT (**a**). The cyst content is homogeneously hyperintense on T2-WI (**b**) and hypointense on T1-WI (**c**). The cyst wall does not show significant enhancement (**d**).

***Cysts***, most often solitary, are usually incidental findings, being asymptomatic [25]. Two categories exist: primary (true) and secondary (false). Primary cysts, also called epidermoid cysts, are congenital lesions with an epithelial lining, with a 20% prevalence in females. Possible explanations for the pathogenesis of true cyst include (1) infolding of peritoneal mesothelium after rupture of the splenic capsule, (2) collections of peritoneal mesothelial cells trapped in splenic sulci, or (3) origin from normal lymph spaces [27]. Primary splenic cysts constitute 10% of all nonparasitic cysts of the spleen. It is a rare condition with an incidence rate of 0.07%, as reported in a review of 42,327 autopsies [28]. Secondary cysts are lined by a fibrous wall and most often are post-traumatic. Rarely they may occur in splenic abscess or splenic infarction [29]. At ultrasound (US), cysts appear as well-defined rounded lesions with a thin wall and anechoic fluid content. They appear homogeneously hypoattenuating on CT and lack enhancement. Calcifications may be seen in the wall in post-traumatic cysts. Unless hemorrhage or debris is present, at MRI, splenic cysts appear hypointense on T1-WI and homogeneously hyperintense on T2-WI (Figure 4). Simple cysts, either primary or secondary, do not warrant further follow-up with imaging. However, due to the increased risk of complications (rupture, infection, hemorrhage), splenic cysts larger than 5 cm or symptomatic ones should be treated surgically, trying to preserve as much of the splenic parenchyma as possible [30].

**Figure 5:**
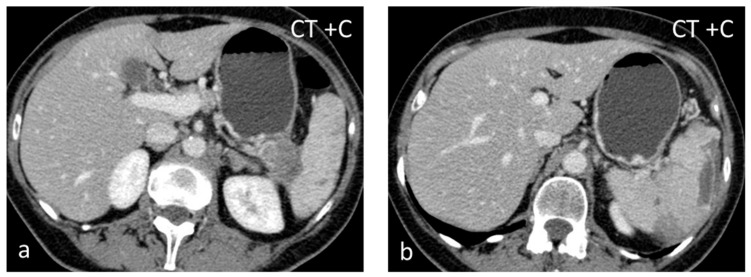
Splenic infarct. Axial CECT images (**a**,**b**) demonstrating peripheral hypoperfused area.

***Splenic infarct*** can be of either arterial or venous origin. Global infarction may be caused by occlusion of the splenic artery. Occlusion of a segmental artery leads to infarction if non-communicating branches are affected. Obstruction can be caused by cardiac emboli or local thrombosis, facilitated by systemic disorders such as vasculitides, hematologic disorders (e.g., sickle cell anemia), leukemia, or lymphoma. There is a great diversity of mechanisms and etiologies for splenic infarction, but it is a rare event with a reported incidence of only 0.016% of admissions to an academic general hospital over 10 years [31]. Acute splenic infarcts usually appear on US as wedge-shaped, peripheral hypoechoic lesions pointing toward the splenic hilum. Over time they become hyperechoic, simulating a pseudolesion; the lack of vascularity at color-Doppler aids in the differential diagnosis. On non-contrast CT, the detection of infarcts is difficult, while after intravenous contrast administration, they typically appear as peripheral, wedge-shaped defects (Figure 5). In the case of global infarction, peripheral contrast enhancement due to collateral flow from capsular vessels can be seen (“rim sign”) [31]. The SI on T1-WI depends on the age of the lesion.

**Figure 6:**
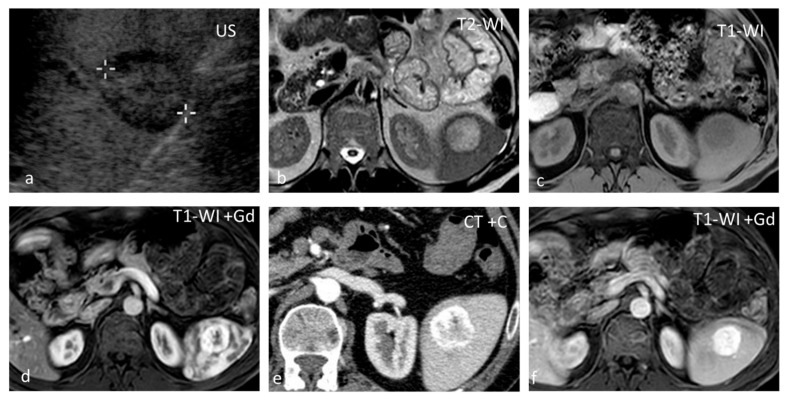
Hemangioma. A well-defined isoechoic splenic lesion with a hypoechoic rim on US (**a**). The lesion appears hyperintense on T2-WI with a thin hypointense rim (**b**) and hypointense on T1-WI (**c**), demonstrating strong peripheral enhancement in the early phases post intravenous contrast administration both at MRI (**d**) and CT (**e**), and homogeneous delayed enhancement (**f**).

***Hemangioma***, although rare, is the most common splenic neoplasm and is found in up to 14% of patients at autopsy. It is formed by a proliferation of vascular channels, lined by a single layer of epithelium, and filled with blood. Hemangiomas are usually asymptomatic, solitary, or multiple. The natural course of hemangiomas is slow growth, and symptoms or complications, when present, occur late. Splenic hemangiomas may occur as part of generalized angiomatosis, as seen in Klippel–Trenaunay syndrome. Complications include rupture, hypersplenism, and malignant degeneration. Kasabach–Merritt syndrome, which involves the triad of anemia, thrombocytopenia, and coagulopathy, has been reported in patients with large hemangiomas [32]. Hemangiomas are round-shaped lesions with well-defined margins and a diameter <2 cm. Calcifications and cystic changes may be seen in up to 30% of cases. On US, hemangiomas have a variable appearance, being most commonly hyperechoic [32]. They appear hypoattenuating on non-contrast CT, hypo- to isointense on T1-WI, and hyperintense on T2-WI (Figure 6). Contrast enhancement can be immediate, homogeneous, and persistent or present as early peripheral enhancement with either uniform delayed enhancement or with fill-in and delayed enhancement of a central fibrous scar. Spontaneous rupture has been reported to occur in 25% of splenic hemangiomas, and treatment in such cases most often consists of splenectomy. In a study including 32 patients with splenic hemangiomas,11 of the patients had splenic lesions characterized as such based on their typical imaging findings on CT and US alone, while all were managed successfully with observation [33].

***Splenic hamartomas*** are very rare splenic lesions, with only around 200 cases reported since 1861 [34]. A review of the autopsy series has shown that the incidence of splenic hamartoma ranges from 0.024% to 0.13%. Hamartomas may occur at any age with equal gender predilection. Most patients have no symptoms, and the discovery of a splenic hamartoma usually is an incidental finding [32]. They present as solid, well-defined, round lesions, which may be associated with tuberous sclerosis. Females usually have larger hamartomas, which may reflect a hormonal influence. Some splenic hamartomas have rapid growth, and larger lesions may rupture [34]. On US, they appear as solid hypoechoic masses; when hemorrhage or cystic changes are present, they can be heterogeneous [35]. Color-Doppler shows rich vascularization. On CT, hamartomas are isodense or hypodense with heterogeneous enhancement. When isodense, they may be identified as focal contour deformities. Fat can be detected as areas with negative attenuation [35]. On MRI, hamartomas appear mildly hypointense to isointense on T1-WI and heterogeneously, mildly to moderately hyperintense on T2-WI, less than haemangiomas. They show diffuse, intense, heterogeneous enhancement in the early arterial phase. They become homogeneously isointense to slightly hyperintense in the delayed phase and reveal a non-enhancing central scar [36]. Although splenic hamartoma should be considered a differential diagnosis for all splenic masses, it is important to distinguish splenic hamartomas from splenic hemangiomas, the most common benign splenic lesions. In addition, despite specific imaging features of splenic hamartomas, it is difficult to rule out the possibility of a malignant neoplasm. Thus, the diagnosis must be confirmed by pathological examination [37].

***Littoral cell angioma*** is another rare vascular tumor that often results in splenomegaly, with less than 150 cases reported in the literature. Due to increasing numbers of littoral cell angiomas described in association with autoimmune disorders (comorbidity rate, 12.2%) and tumors (comorbidity rate, 13.8%), an immune system dysfunction has been postulated as a possible crucial pathogenic mechanism [38,39]. Multiple hypoattenuating nodules of different sizes are seen on CT. When solitary, it is large with prolonged contrast enhancement. It is usually of low signal intensity on both T1 and T2-WI due to its hemosiderin content [38]. The lesion is listed in the differential diagnosis of both benign and malignant lesions; therefore, an imaging diagnosis is rarely obtained [40]. The gold standard treatment is splenectomy, followed by long-term follow-up.

**Figure 7:**
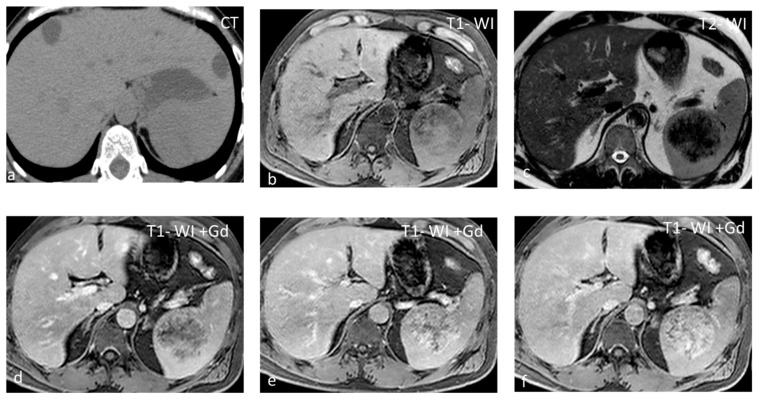
SANT appearing slightly hypodense on non-contrast CT (**a**) and isointense on T1-WI (**b**), with small areas of hyperintensity due to hemorrhage. On T2-WI (**c**), the lesion is heterogeneous, predominantly hypointense, with hyperintense radiating septa. After gadolinium administration, enhancement is progressive (**c**–**f**) along the radiating septa converging toward the center of the lesion.

***Sclerosing Angiomatoid Nodular Transformation (SANT)*** is a rare benign vascular disorder of unknown cause, with the same prevalence as inflammatory pseudotumors and approximately 170 cases described in the scientific literature. SANT is almost exclusively described in the spleen, except for one reported in the adrenal gland [41]. Half of SANTs are asymptomatic, and half are diagnosed because of abdominal pain, pancytopenia, and splenomegaly. SANT is considered a disease of slight female preponderance. The patients usually present in the 30- to 60-year age group. Splenectomy is a useful and effective technique for the management of SANT. SANT patients have a good prognosis, with no recurrence after splenectomy [42]. SANT is formed by multiple coalescing angiomatoid nodules from the red pulp embedded within a dense fibrous stroma [42]. The nodules are constituted by irregular vascular spaces lined by thick endothelial cells; the fibrous bands coalesce to form a central stellate fibrous scar. SANT appears as a well-circumscribed, solitary round mass, typically 3–17 cm in size. It is often mistaken for a sclerosed hemangioma or an inflammatory pseudotumor and can mimic a malignancy [41]. On CT, they present as iso- to hypoattenuating masses, while they appear isointense on T1-WI but can show areas of hyperintensity or susceptibility if a hemorrhage is present. T2-WI shows a heterogeneous, predominantly hypointense mass with hyperintense septa radiating toward the center (Figure 7). Contrast enhancement may be peripheral with radiating lines (spoke-wheel pattern), rim-like, and progressive. In the delayed phase, SANTs appear homogeneous. Moderate and heterogeneous 18F-FDG avidity has also been reported [43].

**Figure 8:**
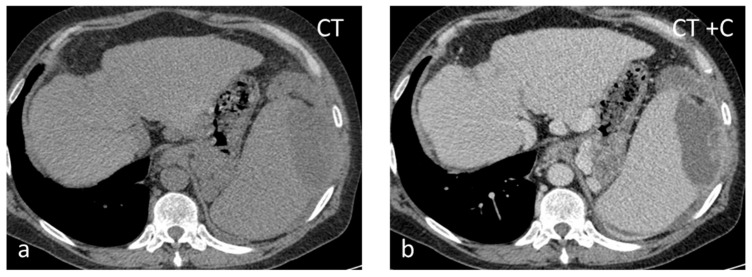
Thirty-seven-year-old male with HIV-HCV infection and splenic abscess from *E. coli*. The abscess appears as an ill-defined, subcapsular lesion with hypodense content on non-contrast CT (**a**) and thick irregular enhancing wall post intravenous contrast administration (**b**).

***Pyogenic abscesses*** are rare (incidence between 0.2 and 0.7% in autopsy series) and frequently unrecognized lesions that can be infective (from hematogenous spread or by direct extension), traumatic, post-infarction, or related to immunodeficiency. They can be solitary or multiple. In the majority of abscesses, streptococcus or staphylococcus is isolated. In the presence of a pyogenic splenic abscess without obvious etiology, it may be helpful to investigate bacterial endocarditis as a possible source of septic emboli [44]. On US, pyogenic abscesses appear as ill-defined hypoechoic or anechoic lesions with debris, fluid levels, and internal septations of varying thickness. Intralesional gas causing echogenic foci with ‘dirty’ shadowing is highly suggestive [45]. CT shows ill-defined lesions with inhomogeneous low attenuation (Figure 8). At MRI, they present with fluid lesion characteristics and show peripheral irregular rim enhancement [46].

***Fungal abscesses*** affect most commonly immunocompromised individuals. The most common infecting organisms are Candida albicans, Aspergillus fumigatus, and Cryptococcus neoformans [45]. Concurrent infection in the liver is very common in patients with splenic candidiasis [44]. The lesions are usually subcentimeter in size and multifocal. On US, fungal abscesses can demonstrate a “bulls-eye” appearance with a central hyperechoic inflammatory core surrounded by hypoechoic fibrotic tissue. CT usually shows multiple small low-attenuation lesions, occasionally with a central hyperdense focus. They have intermediate SI on T1-WI and high SI on T2-WI, with absent or subtle ring-like enhancement [45]. Associated parenchymal infarcts may also be observed in the course of the disease [44].

***Splenic tuberculosis (TB)*** is rare, with few cases reported usually encountered in immunosuppressed individuals, although some case reports have been published on splenic TB in immunocompetent hosts [47]. Splenic TB more frequently occurs as part of a disseminated disease than in an isolated form. The most common symptoms that patients present with are fever (82.3%), fatigue and weight loss (44.12%), and splenomegaly (13.2–100%). Different pathological forms of splenic involvement are described, including miliary tuberculosis, nodular tuberculosis, tuberculous spleen abscess, calcific tuberculosis, and mixed-type tuberculosis [47,48]. Solitary/macronodular TB enters into the differential diagnosis with primary and secondary splenic tumors and with abscesses. Its diameter ranges between 1 and 3 cm. The lesions are hyperechoic in the earlier stage and hypoechoic in the stage of caseating necrosis [26]. On CT, they appear hypodense with a rather heterogeneous enhancement pattern and central irregular necrosis. At a more advanced stage, the lesions can calcify, while associated findings include abdominal lymphadenopathy, high-attenuation ascites with nodular peritoneal thickening, and focal hepatic lesions. Splenectomy is the gold standard not only for diagnosis but also for treatment. In selected cases, a four-drug regimen of anti-tubercular treatment may be an alternative to surgery [49].

***Hydatid cysts*** may occasionally involve the spleen, especially in endemic areas. Isolated hydatid of the spleen is rare, even in endemic regions, constituting 0.8–4% of all cases of human hydatid, while it constitutes 5.8% of all cases of abdominal hydatid. The diagnosis of cystic echinococcosis rests mainly on imaging, with immunodiagnostics playing only an ancillary role due to limitations in sensitivity and specificity [50]. The spleen represents the third most commonly involved organ in the body after the liver and lung [51]. According to a classification initially developed for liver hydatid cysts based on US and later expanded to include CT and other organs such as the spleen, five different types of hydatid cysts are differentiated: type I—univesicular cyst; type II—univesicular cyst with membrane detachment; type III—multivesicular cyst; type IV—pseudotumoral cyst; and type V—fully calcified cyst [52]. In a small series of 10 splenic hydatid cases, four were type I, and three cases each were type II and III, respectively [51]. On US, a mixed pattern of echogenicity may be produced by infolding membranes and dense debris [25]. On CT, this translates into high attenuation, while ring-like calcifications may also be seen in the periphery.

## 4. Conclusions

Benign focal splenic lesions, mostly detected incidentally, are not uncommon. Imaging can often only provide a differential diagnosis rather than a single definite diagnosis. Radiologists should be familiar with the characteristic imaging findings of different benign solitary splenic lesions, which, together with the clinical context, will aid them in giving the correct diagnosis and avoid overtreatment and unnecessary follow-up scans.

## Figures and Tables

**Table 1:** Categories of focal splenic lesions.

Category	Lesion Type
Intermediate between benign and malignant	Inflammatory myofibroblastic tumor
Inflammatory	Sarcoidosis
Congenital/developmental	Lymphangioma Primary cyst
Acquired	False cyst Infarction
Vascular	Haemangioma Hamartoma Littoral cell angioma Sclerosing angiomatous nodular transformation (SANT)
Infectious	Pyogenic abscess Fungal abscess Tuberculosis Hydatid cyst
Malignant	Lymphoma Angiosarcoma Haemangiopericytoma Splenic metastasis

**Table 2:** Cross-sectional imaging characteristics of benign focal splenic lesions.

Lesion	CT	T1-WI	T2-WI	Enhancement
Inflammatory myofibroblastic tumor	LA	↓ SI	↓ SI	slow delayed
Sarcoidosis	LA	↓ SI	↓ SI	minimal delayed
Cyst	LA	↓ SI *	↑ SI	no
Lymphangioma	LA	↓ SI *	↑ SI with hypointense septa	no only septa
Infarct	nv	recent: ↑ SI old: iso/↓ SI	↑ SI	peripheral, wedge-shaped defect
Haemangioma	LA	↓ SI to isointense	↑ SI	variable marked
Hamartoma	LA/isoattenuating	↓ SI to isointense	mildly to moderate ↑ SI	heterogeneous non-enhancing central scar
Littoral cell angioma	LA	isointense	↓ SI	progressive enhancement
SANT	LA/isoattenuating	isointense	↓ SI with hyperintense septa	variable spoke-wheel
Pyogenic abscess	LA	↓ SI	↑ SI	peripheral rim
Fungal abscess	LA	intermediate SI	↑ SI	subtle rim
Tuberculosis	LA	↓ SI	↑ SI	peripheral

LA: Low attenuation. ↑ SI: increased signal intensity (hyperintense). ↓ SI: decreased signal intensity (hypointense). * unless hemorrhagic content/debris/high proteinaceous content. nv: not visible.
